# National Differences in Requirements for Ethical and Competent Authority Approval for a Multinational Vaccine Trial under the EU Directive 2001/20/EC

**DOI:** 10.3390/vaccines3020263

**Published:** 2015-04-14

**Authors:** Eva van Doorn, Eelko Hak, Bob Wilffert

**Affiliations:** 1 Unit of PharmacoEpidemiology and PharmacoEconomics, Department of Pharmacy, University of Groningen, 9713 AV Groningen, The Netherlands; E-Mail: e.hak@rug.nl; 2 Unit of Pharmacotherapy and Pharmaceutical Care, Department of Pharmacy, University of Groningen, 9713 AV Groningen, The Netherlands; E-Mail: b.wilffert@rug.nl; 3 Department of Clinical Pharmacy and Pharmacology, University Groningen, University Medical Center Groningen, 9700 RB Groningen, The Netherlands

**Keywords:** clinical trial, vaccine, directive 2001/20/EC, approval documentation

## Abstract

Obtaining approval for a multinational vaccine trial from an ethics committee and the national competent authority of different Member States of the European Union (EU) is challenging under clinical trial Directive 2001/20/EC because of the differences in the implementation of the directive in national laws of Member States. In this review the national differences in requirements for ethical and competent authority approval are illustrated. The national ethical and competent authority review procedures in Finland, Hungary, The Netherlands, Norway and Slovenia are described under the EU trial directive after discussing the provisions of the trial directive related to both review procedures. The review illustrates the differences between the countries in the documents that have to be submitted for the review procedures, the submission procedures and the language requirements of the documents, the organization of the ethics committees and the role of the competent authority in the approval procedure.

##  1. Introduction

Since 2001, the Directive 2001/20/EC of the European Parliament and the European Council has been regulating the implementation of good clinical practice (GCP) in the conduct of clinical trials on medicinal products for human use [1]. The aim of this directive is to harmonize the national procedures for the ethical approval of clinical trials in Member States of the European Union (EU) [1,2]. Despite the aim of the directive, there has been a severe decline in the number of clinical trials carried out in Europe over the past few years, which according to the European Commission is partly due to the trial directive [3,4]. The directive makes it difficult and expensive to carry out cross-border trials because of the legal form of the directive, since directives from the EU are not directly applicable to Member States [3,4]. Directives are only binding to the result that have to be achieved, which means that Member States have the freedom to choose the form and method to achieve the goal(s) set out in a directive. Therefore, directives are differently implemented in the national law of Member States [5,6,7]. Especially for multinational vaccine trials, such as the EU-funded FLUSECURE and UNISEC projects [8,9], it is a challenge to obtain ethical approval for a trial. These projects need a single opinion from each Member State that will participate in the trial (Article 7 Directive 2001/20/EC) while the procedures to obtain ethical approval differ among the participating Member States [1]. 

On 14 April 2014, the European Council approved a draft regulation on clinical trials on medicinal products for human use and for the repealing of Directive 2001/20/EC [4,10]. This regulation, which is expected to be in effect in 2016 after a start-up period of 2 years, is binding in its entirety and is directly applicable in the Member States [4,5,6,7,10,11]. This means that this type of legislation does not have to be translated or transformed by a Member State before the implementation in the national law. Although the new EU regulation aims to harmonize the national procedure for evaluating clinical trial applications, the ethical review process is still carried out by the concerned Member States. In addition, in case of multistate trials the concerned Member States still have the authority to approve or reject the opinion of the reference Member State [3].

In this review national differences in the requirements for ethical and competent authority approval for a multinational vaccine trial under the EU Directive 2001/20/EC will be illustrated. Therefore, provisions related to the ethical and competent authority review procedure of Directive 2001/20/EC will be described in more detail. The ethical and competent authority approval procedure for a Phase 2 vaccine trial in Finland, Hungary, The Netherlands, Norway and Slovenia will be described as an example, since trial sites in these countries are part of the clinical trial network of the FLUSECURE project [12].

## 2. Directive 2001/20/EC

On 1 May 2001 the EU published Directive 2001/20/EC “*on the approximation of the laws, regulations* *and administrative provisions of the Member States relating to the implementation of good clinical practice in the conduct of clinical trials on medicinal products for human use*” [1]. The directive consists of specific provisions regarding the conduct of clinical trials, including multi-center trials, on human subjects involving medicinal products in the EU (Article 1 (1) Directive 2001/20/EC). Especially provisions relating to the implementation of GCP are included in the directive to provide assurance that the rights, safety and well-being of trial subjects are protected (Article 1 (1,2) Directive 2001/20/EC). Therefore, the directive determines under which terms a clinical trial may be undertaken, e.g., that a clinical trial may only be initiated if the ethics committee has given a favorable opinion and in as much the competent authority of the Member State concerned has not informed the sponsor of any grounds of non-acceptance (Article 9 (1) Directive 2001/20/EC) [1]. The procedures to reach these decisions from the ethics committee and the competent authority may, depending on the sponsor of a trial, run in parallel or not [1]. In addition to the ethical and competent authority approval, other requirement needs to be fulfilled before a clinical trial may be initiated such as the informed consent procedure and that the rights of subjects, to privacy and the protection of data concerning to the subjects, are safeguarded (Article 3 (2) Directive 2001/20/EC) [1].

### 2.1. Ethical Review

Before the start of a trial, an ethics committee has to give a favorable opinion (Article 9 (1) Directive 2001/20/EC). An ethics committee is according to the directive an independent body in a Member State, consisting of healthcare professionals and nonmedical members. They have the responsibility to protect the rights, safety and wellbeing of human subjects involved in a trial and to provide public assurance of that protection by for example expressing an opinion on the trial protocol (Article 2 (k) Directive 2001/20/EC) [1].

To obtain a favorable opinion for a vaccine trial of such a committee, an application has to be submitted before the start of the trial by the sponsor or the principal investigator. In case of multi-center trials, the sponsor or the coordinating investigator (the investigator who is responsible for the coordination of work of the principal investigator and the different trial sites) has to submit the application [13]. According to Article 6 (3) of the directive, an ethics committee should in particular consider specific aspects of an application for a clinical trial in order to establish a reasoned opinion, such as the relevance of the trial and the trial design, the protocol, the quality of the facilities, whether the evaluation of the anticipated benefits and risks is satisfactory and whether the conclusions are justified [1]. However, according to Article 6 (4) of the directive, Member States may decide that the competent authority shall be responsible for the consideration and giving of an opinion on the provisions for indemnity or compensation in the event of injury or death attributable to a trial (Article 6 (3)(h)), any insurance or indemnity to cover the liability of the investigator and sponsor (Article 6 (3)(i)) and the amounts and arrangements for rewarding or compensating investigators and trial subjects, and the relevant aspects of any agreement between the sponsor and the trial site (Article 6 (3)(j)) [1].

An ethics committee should have a maximum of 60 days from the day of receiving a valid (complete) application to give a reasoned opinion about the application to the applicant and the national competent authority (Article 6 (5) Directive 2001/20/EC) [1,13]. Within the time period of the consideration of an application for a favorable opinion, an ethics committee may send a single request for additional information to the applicant. When this is the case, there is a clock stop until the ethics committee has received the additional information (Article 6 (6) Directive 2001/20/EC). Only for trials involving medicinal products for gene therapy or somatic cell therapy or medicinal products containing genetically modified organisms ethics committees may have a longer time period to give a reasoned opinion about the application; 90 days which may be extended by a further 90 days. For trials including medicinal products for xenogenic cell therapy there is no time limit for an ethics committee to give a reasoned opinion (Article 6 (7) Directive 2001/20/EC) [1].

### 2.2. Competent Authority

Besides the submission of a request for an opinion of an ethics committee, a valid request for authorization of a clinical trial has to be submitted to the competent authority of the Member State in which the sponsor plans to conduct the trial before the start of the trial (Article 9 (2) Directive 2001/20/EC) [1]. The reason for this is that a trial may only start if the competent authority has not informed the sponsor of any grounds of non-acceptance [1].

Based on a valid request for authorization, the consideration by the competent authority should take place as rapidly as possible with a maximum of 60 days (Article 9 (4) Directive 2001/20/EC) [1]. If the competent authority of a Member State notifies the sponsor of any ground of non-acceptance, the sponsor may amend the content of a request once only based on the grounds given for non-acceptance. If the competent authority still has grounds for non-acceptance, a trial may not start. However, if the competent authority has not communicated any grounds for non-acceptance to the applicant of the request for authorization the clinical trial may start [1,14]. Nevertheless, for trials with specific medicinal products, written authorization is required prior to the start of the trial. These are clinical trials involving medicinal products for gene therapy, somatic cell therapy including xenogenic cell therapy and all medicinal products containing genetically modified organisms (Article 9 (6) Directive 2001/20/EC) [1,14]. For the consideration of a request of a clinical trial involving these types of products, the competent authority has the same extended time period as ethics committees for the consideration (Article 9 (5) Directive 2001/20/EC) [1]. A Member State may also decide that written authorization is necessary before starting a trial involving medicinal products which do not have a marketing authorization and are referred to in part A of the annex to regulation number 2309/93 of the European Commission [1,14]. These are medicinal products developed by recombinant DNA technology, controlled expression of genes coding for biologically active proteins in prokaryotes and eukaryotes including transformed mammalian cells, or hybridoma and monoclonal antibody methods [15]. Also for other medicinal products with special characteristics it may be decided that written authorization is necessary, such as for medicinal products where the active ingredient or active ingredients is or are a biological product or biological products of human or animal origin (Article 9 (5) Directive 2001/20/EC) [1,14].

## 3. Ethical Approval and Competent Authority Authorization of a Vaccine Trial in Different Member States

Since a directive is not directly applicable in all Member States but is binding to the result that has to be achieved, directives are being implemented differently by national authorities of Member States [5,6,7]. In the EU Member States Finland, Hungary, The Netherlands and Slovenia Directive 2001/20/EC has been implemented in the national law [16,17,18,19]. Norway is not a Member State of the EU, however Norway has a lot of agreements with the EU, e.g., it has joined the European Economic Area (EEA) [20,21,22]. Through the many agreements and other forms of cooperation, Norway has complied with several EU legislations without participating in the process of making the legislative act [23,24,25]. Norway has therefore also fully implemented Directive 2001/20/EC in the Norwegian “*Regulation relating to clinical trials on medicinal products for human use*” [26,27]. Among the Member States, including Norway, there are differences in the overall review procedure of clinical trials such as the organization of the ethics committees and the competent authority, prerequisites before applying for an ethical opinion, the documentation that has to be submitted for ethical and competent authority approval and the submission procedure of the required documents (Table 1).

### 3.1. Ethics Committees

The Member States have implemented the obligation of Article 6 (1) of Directive 2001/20/EC differently to take necessary measures for the establishment and operation of Ethics Committees [1]; all Member States have an ethics committee operating at the national level, and some Member States also have regional and/or institutional ethics committees.

In Finland, there are multiple ethics committees and institutions working in the field of medical research ethics [28,29]. At the national level the National Committee on Medical Research Ethics (TUKIJA) is an expert on research ethics, and it evaluates all proposals for clinical trials on medicinal drugs unless it delegated this task to a regional ethics committee [28,30,31,32,33]. The Finnish regional ethics committees are set up by the board of a hospital district with a university providing medical education in its region (Section 16 “*Medical Research Act*”) [28,29,34].

In Norway the ethics committees for research ethics are also organized at the national and regional levels [35,36]. In contrast with Finland, the regional ethics committees evaluate all concrete medical research projects and have to give approval for medical and health research projects. Norway has seven regional committees for medical research ethics, the Regional Committees for Medical and Health Research Ethics (REC), which are divided based on the geography of Norway [35,36,37,38,39]. The ethics committee at the national level, the National Committee for Medicinal and Health Research ethics (NEM), is an advisory and appealing body for these seven regional ethics committees and therefore does not evaluate proposals for clinical trials as is the case in Finland [35,36,37,38].

The national ethics committees from Hungary and Slovenia review all clinical trial proposals involving medicinal products [19,40,41]. The national ethics committee in Hungary is the Clinical Pharmacology Ethics Committee, in Hungarian known as Klinikai Farmakológiai Etikai Bizottság (KFEB) [17,40,42,43]. The KFEB is one of the national research ethics committees that are part of the Hungarian Health Science Council, which is an advisory board of the Minister of Health [40,44,45]. In Slovenia the National Medical Ethics Committee (NMEC) at the Ministry of Health, which is in Slovene known as Komisija Republike Slovenije za medicinsko etiko, is the ethics committee that gives its opinion about a clinical trial [19,41,46].

**Table 1:** Overview of the differences in the ethical and competent authority approval procedures of a vaccine trial in Finland, Hungary, The Netherlands, Norway and Slovenia.

Aspect Ethical and Competent Authority Approval	Member States				
	Finland	Hungary	The Netherlands	Norway	Slovenia
**Ethics committee**	National (TUKIJA), regional, institution	National (KFEB), institution	Central commission (CCMO), medical research committees (MRECs)	National (NEM), regional (RECs)	National (NMEC)
**Prerequisites before applying for an ethical opinion **	Prior notification TUKIJA	Submit protocol to head of health care institution	-	-	-
**Ethical Review **					
*Documents*	Requirements in the operating procedure of TUKIJA or at the website of the specific regional ethics committee	Requirements in Decree 35/2005 (VIII.26.) of the Minister of Health	Standard research file which is described at the website of the CCMO	Requirements available via the national web-portal at the website of the regional ethics committees	Requirements in the “ *guideline for researchers submitting their projects for ethical review*”
*Submission procedure*	Mail	CD-ROM (three copies)	Preferably digitally on a CD-ROM, signed cover letter and EudraCT form provided on paper	National web-portal	Mail
*Consideration of a request*	60 days	42 days	60 days	60 days	60 days
**Competent authority**	Fimea	OGYI	CCMO or the Minister of Health, Welfare and Sport	NOMA	JAZMP
**Ethical Authorization**					
*Documents*	Requirements in the Fimea “ *Administrative Regulation Clinical Trials on Medicinal Products*”	Same documents as for the ethical review	Same standard research file as for the ethical review	Requirements at the website of the NOMA	Requirements in the “ *Rules on clinical trials on medicinal products*”
*Submission procedure*	Mail	CD-ROM (three copies)	CD-ROM	CD or USB-stick by mail or by e-mail	Mail
*Review procedure*	Assessment content request	Assessment aspects described in Article 13 (3) Decree 35/2005	Marginal review	Assessment content request	Assessment content request
**Ethical Authorization**					
*Consideration of a request*	60 days	60 days	14 days	60 days	60 days
**Language submission procedures**	Review and authorization: Finnish or Swedish, excluding some exceptions	Review and authorization: English, excluding some exceptions	Review and authorization: English, excluding some exceptions	Ethical review: depending on where the trial will be conducted Ethical authorization: English	Review and authorization: English or Slovene, some documents has to be in Slovene
**Other requirements before the start of a vaccine trial**	Batch release by the Fimea for immune-prophylaxis vaccines	Notifications	-	-	-

The ethics committee that has to give a favorable opinion before a scientific research can be conducted in The Netherlands is according to the “*Medical Research Involving Human Subjects Act*” (in Dutch known as “*Wet medisch-wetenschappelijk onderzoek met mensen*” (WMO)), an accredited medical research committee (MREC) or the Central Commission Research Involving Human Subjects (in Dutch known as the Centrale Commissie Mensgebonden Onderzoek (CCMO)) [47,48,49,50,51]. In The Netherlands there are nowadays 24 MRECs, most of them are connected to an academic center or a hospital, which have been accredited by the CCMO [47,52]. Article 2 of the WMO determines in which cases the CCMO has to give a favorable opinion about a scientific research, such as non-therapeutic research involving minors or incapacitated adults (Article 2 (2)(b)(2) and 4 (1) WMO) and scientific research where the assessment by the central commission is desired considering social, legal and ethical aspects of a scientific research (Article 2 (2)(b)(4) WMO) [47,53]. In the decision “*Besluit centrale beoordeling medisch-wetenschappelijk onderzoek met mensen*” (in Dutch), scientific research covered by Article 2 (2)(b)(4) WMO are described [54]. According to Article 1 (e) of this decision scientific research aimed at the development of a vaccine has to be assessed by the CCMO [54].

Only in Finland and Hungary institutional ethics committees play a role in the ethical approval procedure of medical research. The Finnish institutional research ethics committees, which are installed at research institutions, have to give approval for research, which is not covered by the Finnish “*Medical Research Act*” [28,29]. This act applies to all medical research that is defined in Section 2 (1) as research involving intervention in the integrity of a person, human embryo or human fetus for the purpose of increasing knowledge of health, causes, symptoms, diagnosis, treatment and prevention of disease or the nature of diseases in general [34]. Besides, the institution ethics board of the institution that will conduct the trial has to be notified about the trial. Also in Hungary, the Institutional Ethics Committee (IKEB) has to be informed about the admission of a trial by the head of the health institution where the trial will be conducted [17]. The Hungarian IKEBs are installed at each health care institution where research involving human subjects is carried out [40]. In Slovenia there are also a number of regional/local ethics committees at university and regional hospitals, however these ethics committees may not give an independent ethical approval for research projects that have to be reviewed by NMEC [46,55].

### 3.2. Prerequisites before Applying for an Ethical Opinion

Both Finland and Hungary have prerequisites that a sponsor should fulfill before applying for an ethical opinion. The sponsor of a trial should respectively send a notification to TUKIJA or should inform the head of the health care institution conducting the trial [17,31,32,33].

Based on Section 2 (1) of the Finnish “*Decree of the Ministry of Social Affairs and Health on Clinical Drug Trials*” TUKIJA may decide on delegating the handling of a clinical drug trial to a regional ethics committee even before the actual request for an opinion is made [31]. For TUKIJA to be able to make this decision, a prior notification about the clinical trial shall be made to TUKIJA, which is also called a ruling for jurisdiction [31,32]. This prior notification can be applied by the sponsor as soon as it becomes likely that the clinical trial will run in Finland, even if the actual application is not yet complete [33]. The Ministry of Social Affairs and Health has developed a form for the prior notification, the form “Prior notification of a clinical drug trial” (only available in Finnish), that has to be used by the applicant (Section 2.2 Decree on Clinical Drug Trials) [31,33,56]. The completed form needs to be sent by e-mail and mail to TUKIJA, after which TUKIJA will decide if one of the regional ethics committees or TUKIJA will carry out the ethical review of that clinical trial [33].

In Hungary there are some prerequisites before applying for an ethical opinion and authorization [17]. The sponsor of a trial should first submit the Hungarian summary of the protocol to the head of the health care institution conducting the trial. Based on the summary, the head of the health institution should decide whether it accepts the trial in question at the institution in advance for the presence of a future permit form the competent authority. If the head of the health institution admits a trial, the head declares that the health institution is in the possession of all material and personal conditions required for the trial [17]. The form in Annex 1 of Decree 35/2005 (VIII.26.) “*on the clinical trial and application of correct clinical practices of investigational medicinal products intended for human use*”, in which Directive 2001/20/EC has been fully implemented, of the Hungarian Minister of Health can be used for the statement of the head [17,40,57]. If the head admits the clinical trial, the head should inform the competent IKEB on admission of the trial [17]. When the head of the health institution admits the clinical trial, a contract between the health institution and the sponsor of the trial may be concluded [17]. However, this contract is only valid if the clinical trial is officially authorized by the national competent authority (Paragraphs 12–14, Decree 35/2005 (VIII.26.)) [17].

### 3.3. Documents to Be Sent for an Application for a Favorable Opinion

In February 2006 the European Commission published a detailed guidance, based on Article 8 of Directive 2001/20/EC, with the format of the application and the documentation that has to be submitted in an application for a favorable opinion of an ethics committee [1,13]. This guidance, the “*Detailed guidance on the application format and documentation to be submitted in an application for an Ethics Committee opinion on the clinical trial on medicinal products for human use*”, is drawn up in consultation with the Member States and interested parties [1,13]. The guidance is not legally binding; it is aimed to ensure consistent application of the directive. According to this guidance, an application to an ethics committee is valid if all required documents are complete. However, this may differ per Member State of the EU because some Member States ask for specific information [13]. Supplementary Materials Table S1 gives an overview of documents that have to be submitted to the different Member States included in this review based on this detailed guidance and the national provisions of Member States about the ethical submission procedure. 

#### 3.3.1. Documents to Be Submitted to the Ethics Committees in Almost Every Member State

Based on the detailed guidance of the Ethics Committee, and the national provisions of the Member States, the following documents have to be submitted to an ethics committee in almost every Member State:

- A signed cover letter, which according to the detailed guidance should include the European Clinical Trials Database (EudraCT) number, the sponsor protocol number and the title of the trial [13,58]. The cover letter should draw attention to any special issues in the clinical trial such as a special trial population or an unusual trial design. The letter should also specify for each investigational medicinal product (IMP) the reference document(s) chosen by the sponsor to identify the unexpectedness of a serious adverse reaction and should draw attention to any scientific advice or opinion related to the trial or IMP given by the European Medicines Agency (EMA) or concerned Member State or the competent authority or ethics committee of any other country [13].
- An application form signed by the sponsor or the sponsor’s legal representative and/or the coordination investigator [13]. This can be a filled in EU-wide clinical application form (EudraCT application form) or a national or local ethics committee application form [13,59]. For Hungary, The Netherlands and Norway the EU-wide clinical trial application form has to be submitted to the ethics committee [13,17,38,59,60,61,62]. In The Netherlands a national application form (the general assessment and registration (ABR) form) has to be submitted as well, which include questions about the scientific research such as information about the sponsor, the relevance of the scientific research, the risks and benefits of the research and a summary of the research in Dutch and English [63,64,65]. The summary has to be submitted in Dutch and English because both will be published in the CCMO trial register, a public register with all trials, which are registered to run in The Netherlands or carried out by Dutch investigators [64,66]. For the submission for a favorable opinion in Finland, applicants should use the form “*Request for opinion on a clinical drug tria*l” (Section 3 (1) Decree on Clinical Drug Trials) from the Ministry of Social Affairs and Health (not available in English) [31,32,33].
- The clinical trial protocol that describes the objective(s), design, methodology, statistical considerations and organization of the trial (Article 2 (h) Directive 2001/20/EC) [1,13]. The protocol must comply with Chapter 6 of the guidance on GCP (CPMP/ICH/135/95) of the International Conference on Harmonization of Technical Requirements for registration of pharmaceuticals for human use (ICH) and should include [13,67];
  ○ The title of the trial
  ○ The sponsor’s protocol code number
  ○ The number and date of the version that will be updated
  ○ A signature of the sponsor and the coordinating investigator
  ○ All currently authorized amendments
  ○ A definition of the end of the trial
  ○ The evaluation of the anticipated benefits and risks
  ○ A justification of the selection of trial subjects, especially when including subjects who are incapable of giving informed consent or other special populations
  ○ A description of the recruitment and informed consent procedures, especially when subjects who are (temporarily or permanently) incapable of giving informed consent are included or when a procedure with witnesses consent is to be used
  ○ A description of the plan for the provision of any additional care of the subjects once their participation has ended, where it differs from what is normally expected according to the subject’s medical condition

The Hungarian, Norwegian and Slovenian national laws include specific additional information that should be included in the protocol. According to the Hungarian Decree 35/2005 (VIII.26.) the protocol should also include the summary of the protocol in the national language, a peer review of the scientific value of the trial (if available), the exact age of the human subjects and the permission of the director of the hospital [13,17,42]. Also in Slovenia, a summary of the protocol in the national language and the outcome of the peer review of the scientific value of the trial have to be included in the protocol [68]. According to Slovenian “*guideline for researches submitting their projects for ethical review*”, the protocol should also include the aim and scientific rationale, supported by a review of recent literature, and the proposer’s own perception of ethical issues involved in the trial [68]. For the protocol to be submitted for a favorable opinion in Norway, the protocol should include a description of research biobanks that are established in connection with the collection, storage and use of human biological material as part of the research project (Paragraph 25 “*Act on medical and health research*” (Act 20/2008)) [62,69].

- Summary of the protocol in the national language of the Member State [13].
- Information on the IMP in an investigator’s brochure which should consist of all clinical and non-clinical data on the IMP or other products which are relevant to the study of the product or products in human subjects (Article 2 (g) Directive 2001/20/EC) [1,13]. According to the description of the standard research file, which have to be submitted to the Dutch ethics committee, the IMP needs to comply with the provisions in Chapter 7 of the GCP guideline (CPMP/ICH/135/95) [61,67]. Only in Slovenia, the investigator’s brochure does not have to be submitted to the ethics committee based on the “*guideline for researchers submitting their projects for ethical review*” [17,33,61,62,68].
- The arrangements for recruitment of subjects which should include a detailed description of the procedure for enrolment of human subjects, the reasons for the selection of the human subject group and copies of the material that will be used for the recruitment of subjects [13]. In all Member States the arrangements for recruitment of subjects has to be included in the application. However, the description of the arrangement is defined differently among the Member States. For example, for The Netherlands and Norway the application should include the recruitment material that will be used for the recruitment of subjects, whereas Finland defines it as the information on the detailed procedures to be used for the selection of subject [33,61,62].
- The subject information leaflet which consist of all information that will be provided to the subject before their decision to participate in a clinical trial in a language the subjects knows [13]. According to the detailed guidance on Ethics Committees, the information should comply with the elements described under 4.8 in the GCP guideline (CPMP/ICH/135/95) [13,67]. For the submission to the ethics committees in the different Member States, the leaflet should be in the national language of the Member State or according to the Hungarian decree 35/2005 in the mother tongue of the individual or in another language identified by the individual as spoken by him or her [17,33,61,62,63,68,70].
- Form for written consent that should contain at least the consent to participate in the trial, the consent to make confidential personal information available and the consent to archive coded information [13]. This form should also be submitted in the national language of the Member States [17,33,61,62,63,68,70]. The Slovenian “*guideline for researchers submitting their projects for ethical review*” specifies that a separate consent has to be given if biological samples will be collected and part of the material is to be stored for possible future use [68].
- A description of the measures taken to safeguard the subject’s privacy and protection of personal data [13]. Only according to the operating procedure of TUKIJA and the Slovenian “*guideline for researchers submitting their projects for ethical review*” this description has to be submitted to the ethics committee [33,68]. Based on the operating procedure of TUKIJA, a description of the personal data file has to be submitted since this is required based on Section 10 of the “*Finnish Personal Data Act*” (No. 523/1999) [33]. The section of this act determines that the person for whom a personal file is set up and who can determine the use of the file (the data controller), shall draw up a description of the personal data file [71]. This description should contain the name and address of the data controller, the purpose of processing personal data, a description of the group of data subjects and data groups, the regular destinations of the data, whether the data are transferred to countries outside the EU or the EEA and a description of the principles in accordance to which the data file is secured [71]. For the application for a favorable opinion of the NMEC in Slovenia, a description of the arrangements for confidentiality of personal data and the right to privacy, as well as a description whether the human subjects that participate in the trial will have access to the results on their health and the general outcome, should be included [68].
- *Curriculum vitae* and/or other relevant documents of the principal investigator to determine the qualification of the principal investigator [13]. According to the detailed guidance on Ethics Committees, a description of any previous training in the principles of GCP or experience obtained from work with clinical trials and patient care should be described [13]. For the application to the ethics committee in Hungary, The Netherlands and Norway the *curriculum vitae* of the principal investigator(s) should be submitted [17,61,62]. Whereas for Finland and Slovenia no *curriculum vitae* of the principal investigator has to be submitted [33,68]. In Finland a statement on the aptitude of the researches in charge of the proposed trial and the investigators based on other trial sites should be included [33]. For Slovenia a statement of the head of the institution must be included that states that the responsible researchers are capable of recognizing dangerous adverse events and take appropriate care of any clinical contingency [68].
- A description of facilities of the trial so that an ethics committee can give an opinion on the quality of the facilities [13]. For the application to the ethics committee in Finland a statement by the researcher in charge of the proposed trial regarding the quality of the trial facilities and the available equipment has to be included [33]. The submission for The Netherlands should include a statement on the feasibility of the research in a Dutch (investigation) center from the head of the department or the healthcare group manager, and in Hungary a description of the trial facilities has to be submitted, along with information on the supporting staff [17,61].
- In Finland, The Netherlands, Norway and Slovenia a description of the provisions for indemnity or compensation in case of injury or death of trial subjects should be submitted to the ethics committee [13,33,61,62,68]. In Slovenia and The Netherlands, information about the compensation and insurance in case of injury or death, respectively, should be submitted [61,68]. For The Netherlands this should be the insurance certificate for WMO research [60,61]. For Finland the insurance should cover for potential subjects in cases where patients insurance and pharmaceutical injuries insurance do not cover the trial, and in Norway the manufacture or the principal investigator should be a member of the Norwegian Drug Insurance Association [27,33,72,73]. Only the Hungarian national provisions do not include that a specific description of the provisions of indemnity or compensation in case of injury or death of trial subjects has to be submitted [17].
- A description of any insurance or indemnity to cover the liability of the sponsor and the investigator has to be submitted to the ethics committees in Hungary, The Netherlands and Slovenia [13,17,60,61,68]. For the application for a favorable opinion in The Netherlands, proof of coverage of the liability of the investigator or the sponsor has to be submitted and in Slovenia information about the insurance policy [60,61,68]. For the application to an ethics committee in Hungary, any insurance or indemnity to cover the liability of only the sponsor should be submitted [17]. According to Section 3 (5) of the “*act on Medicinal Products for Human Use and on the Amendment of Other Regulations Related to Medicinal Products*” (Act XVC/2005) the sponsor should obtain sufficient liability insurance that covers any damages that may occur in connection with the clinical trial from an insurance company that is establish or has a branch in a Member State of the EEA [74]. Only in Norway the conformation of a signed insurance has to be submitted to the competent authority [1,26]. In Finland the description of any insurance or indemnity to cover the liability of the sponsor and the investigator is not included in the list of required documents to be submitted to an ethics committee in the operating procedure of TUKIJA nor in the list of required documents to be submitted to the competent authority which is included in the administrative regulation of the competent authority [33,75].
- In all Member States information about the financial arrangements between the sponsor and the subjects and/or investigators in the trial has to be submitted to the ethics committees [13,17,33,60,61,62,68]. For Finland the trial fees and remuneration have to be included, in Slovenia the information about the compensation/financial award to the investigators and the supporting staff, and in Hungary information about the compensation of the trials subjects [17,33,68]. In Hungry the cost of the clinical trial also has to be provided including the division of costs between the hospital and the investigator [17]. For the submission of a request to the ethics committee in The Netherlands, information about the financial compensation for the human subjects, the investigators and participating centers, such as the contract between the sponsor, the investigator and the participating center, only has to be provided if the information on the ABR-form is not sufficient [60,61]. Also in Norway a description of the compensation of the institution, any fees paid to patients and/or researchers and the compensation of participants should be included in the application [62].

#### 3.3.2. Specific Required Documents for a Valid Request for Ethical Approval in the Different Member States

The different Member States request specific additional information besides the documents that have to be submitted to almost every Member State (Supplementary Materials Table S1). For example in Finland, Hungary and Slovenia, a statement by the principal investigator and/or the head of the institution where the study is to be conducted has to be submitted regarding the conformity of the trial to research ethics [17,33,68]. However, the content of such statement differs among the Member States. In Finland this statement regarding the conformity of the trial with research ethics should especially focus on the appropriateness of the trial’s aims and planning and the evaluation of the risk and benefits [33]. While for Hungary a letter of intent by the principal investigator should be enclosed in which the investigator agrees to execute the protocol in compliance with GCP principles, the stipulations of the national competent authority decision and the opinion of KFEB in collaboration with the competent IKEB of the investigation location [17]. Whereas for Slovenia a statement of the head of the institution or department where the study is to be conducted has to be included along with a statement of the principal investigator. In these statements the head and the principal investigator need to declare to adhere to the principles of the declaration of Helsinki, the Oviedo Convention on Human Rights and Biomedicine and the Slovene Code of Medical Deontology [68].

Especially for the application for a favorable opinion to an ethics committee in The Netherlands and Hungary additional information is required. Here the documents that have to be sent to the ethics committee are the same as those that have to be sent to the competent authority based on Article 2 “*Regeling wetenschappelijk onderzoek met geneesmiddelen*” and Paragraph 13 (1,4) of Decree 35/2005 (VIII.26.), respectively [17,60]. For example, in both Member States a copy of the importer authorization and examples of the labels in the national language have to be submitted to the ethics committee [17,61]. However, the additional information that has to be submitted to Hungary and The Netherlands is not identical. In Hungary an application should for example also include an analytical certificate of the IMP and, if applicable, a Transmitting Animal Spongiform Encephalopathy (TSE) certificate, which is needed for products of specific animal species, such as sheep and goats [17,76,77]. Products of these animal species need to comply with the provisions in the “*Note for Guidance on Minimising the Risks of Transmitting Animal Spongiform Encephalopathy via Medicinal Products*” from the European Commission because TSE may cause diseases in animals, like BSE, and human subjects, such as Creutzfeldt-Jakob Disease [76,77]. Also for The Netherlands specific information has to be submitted such as a trading license if the IMPs are stored and are not intended for immediate use in a clinical research organization. The reason for this is that only persons with this license may store IMPs [61]. The standard research file should also include the composition and charter of the Data Safety and Monitoring Board (DSMB) [61]. According to the “*Guideline on Data Monitoring Committees*” of the EMA, the sponsor of a clinical trial should, in the planning phase of a trial, assess the need of a DSMB based on the study population, study endpoint(s), the indication, duration of the trial and the available knowledge about a medicinal product [78]. This guideline also mentions situations in which a DSMB might be necessary, such as a trial in life-threatening diseases, and situations where a DSMB might not add or does not add much to a research, such as clinical trials than can be performed in a short time frame [78]. If a DSMB will be installed, the composition of the DSMB needs to be provided in the standard research file along with a signed charter, which consists of information about the DSMB such as the role and the responsibilities of the DSMB and the organization of the DSMB meetings [61,79].

### 3.4. Submission Procedure of the Application for a Favorable Opinion to the Ethics Committees

The submission procedure of the documentation that is required for an ethics committee to make a decision about a clinical trial differs among the Member States. In both Finland and Slovenia the required documents have to be sent by mail to the national ethics committees [33,68]. If one of the Finnish regional ethics committees is the ethics committee that has to give an opinion, the submission procedure should be checked on the website of the specific regional ethics committee. In The Netherlands the submission procedure of a vaccine trial to the CCMO should preferably be done digitally on a CD-ROM [80]. However, the cover letter and the EudraCT application form should be signed by the applicant and provided on paper [63,80]. Also for Hungary the documents should be provided on a CD-ROM. An application for a vaccine trial needs to be sent by the sponsor in three copies on a CD-ROM to the national competent authority of Hungary, Országos Gyógyszerészeti Intézet (OGYI) (Paragraph 13 (1,4) Decree 35/2005 (VIII.26.) [17,42]. One of the copies will be sent to KFEB and one to the National Epidemiological Centre, in Hungarian known as Országos Epidemiológiai Központ (OEK) [17,42,81]. The OEK has an advisory function on biological products, such as vaccines, and is, according to Paragraph 13 (4) of Decree 35/2005 (VIII.26.), a specialist authority, which will assist OGYI in the assessment of the application for an official authorization of a clinical trial [17,81]. The application for a prior approval to a REC in Norway needs to be electronically submitted via the national web-portal at the website of the RECs [82]. Based on the information on this web-portal (which only can be accessed after creating a user account on the REC’s website), the person who needs to create and submit the application form for the prior approval is the chief investigator [62]. For clinical trials, the principal investigator in Norway has to be the chief investigator. The chief investigator may give permission to others to complete the application as a co-user, nevertheless the chief investigator is the person that has to submit the application. Information that needs to be filled in on the application form consists of general information about the trial, such as the Norwegian title of the trial and the institution responsible for the research, and more specific information, such as a project description, the research method and the insurance for research for human subjects [62]. As soon as the chief investigator has submitted the application form via the web-portal, the portal will allocate the review to one of the seven RECs. In general, a project will be transferred to a REC in the region of origin of a research project [82].

### 3.5. Consideration of a Request for Ethical Approval by the Ethics Committees

The ethics committees in the different Member States will consider certain aspects of a request for ethical approval. These aspects, which are described in the national legislations of Finland, Hungary, The Netherlands and Norway, comply mostly with the aspects described in Article 6 (3) of Directive 2001/20/EC [1,17,27,34,47]. Only the ethics committee in Finland does not give an opinion about any insurance or indemnity to cover the liability of the investigator and the sponsor (Article 6 (3)(i) Directive 2001/20/EC) based on Section 10d of the Finnish “*Medical Research Act*” and the ethics committee in Hungary does not give an opinion about the provision for indemnity or compensation in the event of injury or death attributable to a clinical trial based on Paragraph 17 (2) of Decree 35/2005 (VIII.26.) [17,34]. The ethics committee in Hungary should also consider certain aspects, which are not included in Article 6 (3) of Directive 2001/20/EC such as the justification of the application of a placebo group [1,17]. For Slovenia it is possible that the aspects to be considered by an ethics committee are described in a specific national law, however all national legislative acts of Slovenia are only available in Slovene and not in another language [83,84].

All Member States comply with Article 6 (5) of Directive 2001/20/EC which determines that an ethics committee should have a maximum of 60 days from the date of receipt of a valid application for a favorable opinion to give its reasoned opinion [1,17,19,27,34,47]. In all Member States, except for Hungary, the ethics committee that has to give an opinion should, according to the national legislations, give its opinion to the applicant within 60 days from the day of receiving a valid request [17,19,27,34,42,47]. Section 10d of the Finnish “*Medical Research Act*” and Section 3.3 of the Norwegian *“Regulation relating to clinical trials on medicinal products for human use*” also specify that within this time period the ethics committee should submit the ethical opinion to the national competent authority [27,34]. However, in the national legislative act of The Netherlands and the Slovenian “*Medicinal Products Acts*”, which could be found in an unofficial consolidated text in English, it is not specified that the ethics committee also has to submit its opinion to the national competent authority [19,47,85]. Only in Hungary does the ethics committee have a time period shorter than 60 days to give a reasoned opinion. The KFEB, which receives one of the three copies of an application from OGYI, will send its professional-ethical opinion within 42 days to OGYI from the day of receiving the application from OGYI (paragraph 17 (1,5,7) decree 35/2005 (VIII.26.) [17,42].

### 3.6. Competent Authorities

In all Member States the national competent authority has to authorize a trial before the start of the trial (Article 9 (2) Directive 2001/20/EC) [1,17,19,27,47,86]. In Finland, Hungary, Norway and Slovenia there is one national competent authority involved in the authorization of clinical trials, receptively the Finnish Medicines Agency (Fimea) [34,86], OGYI [17,42], the Norwegian Medicines Agency (NOMA) which is in Norway known as Statens legmiddelverk [27,87], and the Agency for Medicinal Products and Medical Devices of the Republic of Slovenia which is in Slovenia known as Javna agencija Republike Slovenije za zdravila in medicinske pripomočke (JAZMP) [19,88,89]. In The Netherlands the CCMO or the Minister of Health Welfare and Sport is the competent authority that has to authorize a trial. If a MREC is the commission that has to give a favorable opinion about a scientific research with medicinal products, the CCMO is the competent authority. When the CCMO has to give an opinion, such as for vaccine trials, the Minister of Health, Welfare and Sport is the competent authority (article 13i (1,5) WMO) [47].

### 3.7. Documents to Be Sent for Competent Authority Approval 

To be able to authorize a clinical trial, a valid request for authorization of a clinical trial has to be submitted to the competent authority. For the request for authorization the European Commission has also developed a detailed guidance drawn up in consultation with the Member States. This guidance, “*Detailed guidance on the request to the competent authorities of a clinical trial on a medicinal product for human use, the notification of substantial amendments and the declaration of the end of the trial (CT-1)*”, is drawn up based on Article 9 (1) of Directive 2001/20/EC [1,59]. The guidance describes the format and content of the request to the competent authority and which documentation should be included in the request [1,59]. Supplementary Materials Table S2 gives an overview of the documents that have to be submitted to the competent authorities of Finland, Hungary, The Netherlands, Norway and Slovenia for a request for authorization based on this detailed guidance and the national legislations of the Member States.

#### 3.7.1. Documents to Be Submitted to the Competent Authority in Almost Every Member State

For the request for authorization by the national competent authority in the different Member States, the following documents have to be submitted in almost every Member State;

- A signed cover letter by the applicant, which, according to the detailed guidance CT-1, should, as with the ethics committee, include the EudraCT number, the sponsor protocol number and title of the trial and should draw attention to any special issues of the trial [13,59]. The cover letter for the application to the competent authority in Finland should also include the name of the person responsible for the trial in Finland [75]. Since, according to the “*Finnish Medicines Agency Administrative Regulation Clinical Trials on Medicinal Products*” (2/2012), a foreign sponsor must have a representative in Finland who is responsible for communication with the Fimea [75]. The cover letter which has to be submitted to the Slovenian competent authority, JAZMP, should be in Slovenian according to article 20(2) of the “*Rules on clinical trials on medicinal products*” [90,91].
- A filled in and signed EU-wide clinical application form (EudraCT form) [59].
- The signed protocol of the clinical trial that should according to detailed guidance CT-1 comply with the content and format described in Chapter 6 of the GCP guideline (CPMP/ICH/135/95). A protocol for a multicenter trial should be signed by the sponsor and the overall coordinating investigator (2.5.52. Detailed guidance CT-1) and should in particular include [59]:
  ○ The title of the trial
  ○ The sponsor protocol number specific for all versions
  ○ The date and number of the version that will be updated when it is amended, and a short title or name assigned to it
  ○ A clear and unambiguous definition of the end of the trial
  ○ A description of the plan for the provision of any additional care for the trial participants once their participation in the trial has ended, where it differs from what is normally expected according to the medical condition of the clinical trial participant
  ○ A clear address to all sub-studies conducted at all trial sites or only a specific site
  ○ A discussion of the relevance of the clinical trial and its design
  ○ An evaluation of the anticipated benefits and risks
  ○ A justification for including participants who are incapable of giving informed consent or other special populations such as minors
  ○ A detailed description of the recruitment and informed consent procedure, especially when participants are incapable of giving informed consent
  ○ An identification of adverse events or laboratory anomalies critical to safety evaluations
  ○ A synopsis of the protocol

These aspects correspond mostly with the aspects, which in particular should be included in the protocol for the request of a favorable opinion of an ethics committee [1,59]. Only the discussion of the relevance of the clinical trial and its design, addressing sub-studies conducted at all trial sites or only a specific site, the identification of adverse events or laboratory anomalies critical to safety evaluation, and a synopsis of the protocol are not stated in the detailed guidance on Ethics Committees [1,59]. For the submission of the protocol to the Fimea, the protocol also has to be signed by the person who is responsible for the clinical trial in Finland. Chapter 8 of the regulation of the Fimea summarizes which specific information should be included in the trial protocol, such as the purpose of the investigation and a description of patients [75].

- The investigator’s brochure [59].
- Based on the detailed guidance on competent authorities, an IMP dossier (IMPD) or non-investigational medicinal product (NIMP) dossier has to be submitted [59]. Where an IMP is a pharmaceutical form of an active substance or placebo being tested or used as a reference in a clinical trial including products already with a marketing authorization but used or assembled in a way different from the authorized form, for an unauthorized indication, or used to gain further information about the authorization form (Article 2 (d) Directive 2001/20/EC) [1]. For example vaccines tested in trials, which are intended to evaluate the safety and efficacy of the vaccines [1,92]. Therefore, for all Member States an IMPD has to be submitted to the national competent authority for the authorization of a vaccine trial [17,26,61,75,91]. An IMPD should give information about the quality of the IMP, such as the chemical and pharmaceutical quality, manufacturing and control of the product, non-clinical pharmacology and toxicology data, and clinical trials and human experience data [59].
- A copy of the opinion of the ethics committee of the Member State concerned has to be submitted to the competent authority in Hungary, Norway and Slovenia [17,26,59,91]. According to the detailed guidance CT-1, a copy of the opinion of the ethics committee has to be submitted as soon as it is available, unless the ethics committee informs the applicant that is has copied its opinion to the national competent authority of the Member State [59]. In The Netherlands a copy of the assessment of the ethics committees or competent authorities from other Member States of the EU has to be included in the standard research file [61]. Only for Finland it is not specified in the national regulation about the application to the competent authority that a copy of the ethics committee that has to give an opinion about a trial has to be submitted to the competent authority [75].
- The content of the labeling of the IMP which should be in the official language(s) of the Member State according to Article 14 of Directive 2001/20/EC [1,59]. For all Member States, except for Finland, it is included in the national information about the request for authorization that examples of the labels have to be submitted in the national language [17,26,61,75,91]. The Netherlands, Norway and Slovenia specify that the labels should comply with the requirements in Annex 13, under Point 16, of the directive “*laying down the principles and guidelines of good manufacturing practice in respect of medicinal products for human use and investigational medicinal products for human use*” of the European Commission (Directive 2003/94/EC [26,61,91,93,94].
- The subject information leaflet. This leaflet is not included in the detailed guidance CT-1 but has to be submitted to the competent authorities in Finland, Hungary, The Netherlands and Slovenia based on respectively the regulation of the Fimea [75], decree 35/2005 [17], the explanatory notes on the standard research file [61], and the Slovenian “*Rules on clinical trials on medicinal products*” [90,91].
- 45) The informed consent form, which is also not included in the detailed guidance CT-1 but in the national information of Finland, Hungary, The Netherlands and Slovenia [17,59,61,75,91].
- For the submission to the competent authorities in Hungary, The Netherlands and Slovenia a summary of the protocol in the national language needs to be included [42,63,64,65,91]. In Hungary, this summary has to be included in the protocol [42], in The Netherlands with the ABR-from [63,64,65], and for Slovenia five copies of the summary of the trial have to be submitted to the competent authority in Slovenian language [91].

#### 3.7.2. Additional Required Documents for a Request for Competent Authority Approval

According to the detailed guidance on competent authorities, additional documents have to be submitted besides the cover letter, application form, protocol, investigator’s brochure and the IMPD, such as the copy of the opinion of the ethics committee of the Member State concerned and the content of the labeling. The detailed guidance also includes other additional documents, which have to be provided to the national competent authority: a copy of the summary of scientific advice from any Member State or the EMA with regard to the clinical trial, a copy of the EMA’s decision on the agreement of the Paediatric Investigation Plan and the opinion of the Paediatric Committee if the clinical trial is part of an agree pediatric investigation plan, and proof of payment in case of fees [59]. Only The Netherlands and Norway have specified that copies of the assessment by other regulatory authorities should be submitted to the competent authority [26,61]. Besides, Norway is the only Member State, which specified that, if applicable, the final summary report for a pediatric investigational plan has to be submitted to the competent authority [26]. This information is based on the website of the competent authority since the guideline to the Norwegian regulation to clinical trials, which according to the competent authority specifies the content of a request for authorization, is only available in Norwegian [26,95,96]. Furthermore, proof of payment in case of fees only has to be submitted to the competent authorities in Finland and Slovenia [75,91]. In Finland 2200 Euros have to be paid for the clinical trial notification to the Fimea [75,97]. However, if a clinical trial will be conducted without external financing or with financing by a non-profit corporation, a waiver of processing fee may be requested. The notification of the Fimea should then be accompanied by an informal statement to the effect that the investigation will not receive any outside financing [75,97]. For the notification or request for authorization of a clinical trial in Slovenia 750 or 1500 Euros, respectively, have to be paid [91,98]. According to Article 52 and 62 of the Slovenian “*Medicinal Products Acts*” the Slovenian competent authority JAZMP has to give written authorization for clinical trials involving human medicinal products for gene, therapy, somatic cell therapy, including xenogenic therapy and all medicinal products containing genetically modified organisms. For clinical trials involving other medicinal products, the competent authority only needs to be notified [19].

The national competent authorities may also ask for additional documentation to the documentation listed in the CT-1 guidance because Member States can have decided that the competent authority is responsible for the consideration of aspects described under Article 6 (3)(h,i,j) of Directive 2001/20/EC [1,59]. Additionally, Member States could have national provisions on the protection of clinical trial subjects that are broader than the provisions of Directive 2001/20/EC (2.10 detailed guidance) [59]. However, according to the detailed guidance CT-1 competent authorities may only ask for additional documentation and information if the national provisions are in line with Directive 2001/20/EC and if it is appropriate and proportionate considering the aim of the provision [59].

### 3.8. Submission Procedure to the Competent Authorities

In Finland, Hungary and Slovenia the submission procedure of the documents that are required for a request for competent authority approval are the same as for the submission procedure to the ethics committee in these Member States [42,90,99]. However, the filled in EudraCT application form should also be submitted electronically as an XML-file on a CD-ROM in Finland and Slovenia [42,59,75,91,99]. For the submission procedure to the competent authority in The Netherlands all documents should be provided on a CD-ROM [80], and in Norway the documents have to be sent on a CD-ROM or USB‑stick by mail or by e-mail [26]. In The Netherlands the XML-file of the EudraCT form does not have to be sent with the standard research file [61].

### 3.9. Review Procedures Competent Authorities

In all Member States, except for The Netherlands, the national competent authority reviews the content of a request for competent authority approval. Paragraph 13(3) of the Hungarian Decree 35/2005 (VIII.26.) on the clinical trial and application of correct clinical practices has specified the aspects of an application that OGYI will assess. These include the clinical trials conducted with the trial preparation, whether the protocol meets the professional standards, whether the expected risks are surpassed by the expected therapeutic benefits and whether the IMP can be administrated to humans [17]. The decision of OGYI can only be adopted after receiving the professional-ethical opinion of the ethics committee KFEB; OGYI may not authorize a trial if KFEB rejects the application on professional and ethical grounds (Paragraph 14 (1) Decree 35/2005 (VIII.26.) [17,42]. OGYI can also only authorize the use of an IMP on humans if the personal conditions for the head of the clinical trial and the material conditions of the trial site meet the requirements in Annex 2 of Decree 35/2005 (VIII.26.) [17]. In The Netherlands, the assessment by the competent authority is a marginal review [100,101]. The CCMO or the Minister of Health, Welfare and Sport only checks the European databank with adverse events (EudraVigilance) as to whether the database includes side effects of the IMP which lead to unacceptable risks to the human subjects or if there is otherwise evidence that the research will lead to risks to the human subjects of the research (Article 13j (1) WMO) [47,100,101]. The assessment of the content of the request is done by the commission that has to give a favorable opinion [101,102].

### 3.10. Consideration of a Request by the Competent Authorities

All Member States comply with Article 9 (4) of Directive 2001/20/EC, which determines that the consideration of a request for authorization may not exceed 60 days [1,17,19,27,47,86]. Based on the national law of Finland, Norway and Slovenia, a vaccine trial may start if the Fimea, NOMA or JAZMP, respectively, has not responded to the applicant about its assessment within 60 days from the day of receiving a valid request for competent authority approval [19,27,86]. Only for clinical trials involving specific medicinal products is written authorization required in these Member States. In Norway these are clinical trials involving medicinal products described under Articles 9 (5) and 9 (6) of the EU trial directive, and in both Finland and Slovenia clinical trials involving medicinal products described under Article 9 (6) of Directive 2001/20/EC [1,19,27,86]. For Hungary, the competent authority OGYI can adopt its decision on a clinical trial if the KFEB has given a favorable professional-ethical opinion [17,42]. Based on Paragraphs 14 (3) and (4) of the Hungarian Decree 35/2005 (VIII.26.), OGYI should send the authorization decision of a clinical trial, along with the professional-ethical opinion of the KFEB, to the sponsor within 60 days from the day of receiving an application for an authorization [17]. OGYI should also send the authorization decision to KFEB and the National Pension Insurance Fund according to Paragraph 14 (3) [17]. Only The Netherlands have laid down a shorter period than 60 days in their national law. Based on Article 13i (1,3) WMO, a vaccine trial may start if the Minister of Health, Welfare and Sport has not communicated reasoned objections for the research within 14 days from the day of receiving an application [47]. However, just like Finland, Norway and Slovenia, for clinical trials involving medicinal products described under Article 9 (6) of Directive 2001/20/EC (Article 13i (5) WMO), written authorization is required [47].

### 3.11. Language Requirements Submission Procedures

For the submission of the required documents for a request for a favorable opinion by an ethics committee and the competent authority approval there are differences in the language requirements among the Member States. In both Hungary and The Netherlands the documents for the request for ethical and competent authority approval may be submitted in English [17,42,61,63]. Furthermore, in Slovenia the documents that have to be submitted for a request for ethical approval may also be submitted in English to NMEC. However, the documents may also be submitted in Slovene language [68]. In all three Member States, the summary of the protocol, examples of the labels and the information that will be provided to the human subjects such as the informed consent form and diaries need to be provided in the national language [17,42,61,63,68]. For Slovenia, the statement that appropriate measures will be taken to prevent pregnancy in case of research involving patients in fertile period where risk of mutagenicity exists also needs to be submitted in Slovene language [68]. There is one exception for The Netherlands, the questionnaire that must be completed by participants may be submitted in English if the English questionnaire is usually used in The Netherlands and has been validated [61]. The language requirement for the documents that have to be submitted for a request for competent authority approval by JAZMP is not specified in the “*Rules on clinical trials on medicinal products*” with the exception that the cover letter and the summary of the trial protocol should be submitted in Slovenian [90,91]. 

In Norway the request for competent authority approval by the NOMA may be submitted in English, but the language requirement for the documents to be submitted for the request for ethical approval is dependent on where the clinical trial will be conducted [62,95]. Only for research projects that are exclusively conducted in countries other than Norway, the application for the prior approval by a REC may be submitted in English. If a project is not exclusively conducted in countries other than Norway, the application form and the participant information should be written in Norwegian. The attachments other than the participant information may in this case be submitted in English or another Scandinavian language [62]. Ethics committees in Finland do not accept a request for an ethical opinion in English [13,33]. Therefore a foreign sponsor must have a representative in Finland who is responsible for the communication with TUKIJA or the regional ethics committee and also with the Fimea [13,33,75]. Only the trial protocol and investigator’s brochure may be submitted in English to the ethics committee. The summary of the trial protocol, information for potential subjects and informed consent form needs to be translated in both Finnish and Swedish [33,70].

### 3.12. Start of a Vaccine Trial

In all Member States a vaccine trial may start if the competent ethics committee has given a favorable opinion and if the competent authority has not responded to the applicant within the defined time period from the day of receiving a valid request for authorization or, depending on the nature of the vaccine, has given written authorization [17,19,27,47,86]. This corresponds with Article 9 (1) of Directive 2001/20/EC [1]. Only in Hungary, will the decision of the national competent authority OGYI always be sent to the applicant [17]. The reason for this is that before the start of a trial in Hungary, the sponsor should have sent the decision of OGYI along with a summary of the protocol in Hungarian language to the competent IKEB (Paragraph 14 (5) Decree 35/2005 (VIII.26.)). The task of the IKEB is to protect the rights and safety of the human subjects participating in the trial and to monitor the execution of the trial in accordance with the conditions in the protocol, the official authorization of OGYI and the opinion of KFEB (Paragraph 12 (4) Decree 35/2005 (VIII.26.)). The sponsor also has to notify the head of the health institution and the principal investigator about the decision of OGYI (Paragraph 14 (3) Decree 35/2005 (VIII.26.)). Besides, the sponsor also has to inform the investigators about the professional-ethical opinion of the KFEB (Paragraph 14 (4) Decree 35/2005 (VIII.26.)) [17].

For vaccine trials with immunoprophylaxis vaccines in Finland, the Fimea needs to release the batches of the vaccines before the start of a trial. The reason for this is that without the authorization of the Fimea these batches may not be used [75]. To be able to release the batches, the applicant should send the manufactures own analysis certificates to the Fimea along with the relevant batch release certificates signed by the qualified person, details of the batch quantities to be imported into Finland, details on the person responsible for the trial and the clinical trial authorization granted by the Fimea [75]. The Slovenian “*Medicinal Products Acts*” also includes a provision about the import of vaccines into Slovenia, namely Article 77 (2). According to this article the entry and import of vaccines into Slovenia is only allowed on the basis of an entry or import authorization that needs to be given by the competent authority [19]. However, according to the competent authority, no special or additional import permit is required for any kind of drugs, including vaccines, used in clinical trials. This exception is, according to JAZMP, based on the exceptions of Article 77 (2) of the “*Medicinal Products Act*” which are described in the “*Rules on the conditions, method and procedure for the acquisition of authorisation for entry or import of medicinal products for human use*”. However, these rules are also only available in Slovenian language [103].

## 4. Conclusions

Under the EU clinical trial Directive 2001/20/EC it is a challenge to obtain ethical approval for a multinational vaccine trial from each Member State of the EU participating in the trial as illustrated by this review due to the differences in the implementation of the directive in the national laws of the Member States. There are differences between the Member States in the documents that have to be submitted to the ethics committee and the national competent authority, which has to perform ethical approval of the trial, the submission procedure of the documents and the language requirements. Moreover, there are differences between the Member States in the organization of the ethics committees and the role and position of the competent authority in the procedure of the approval of a trial. Especially for the requirements of the documents that have to be submitted, including the language requirements of these documents, and the submission procedure a more harmonized procedure in the Member States is desirable for sponsors of multinational trials. The aim of this is to simplify the process of obtaining ethical and competent authority approval in different participating Member States for the start of a trial. For the new EU trial regulation to harmonize the procedures and to be in effect in 2016, much work has to be done, since Member States have to adapt their procedures to the new regulation which is binding in its entirety. Therefore, it is advisable that the implementation of the new trial regulation in the national law of Member States and the changes in the organization of the national review procedures will be carried out with high priority in close collaboration of the Member States and interested parties, with the aim of making an effective transition process from the EU trial directive to the new EU trial regulation possible in all Member States, which indeed harmonizes the national procedures of the Member States.

## Supplementary Files

Supplementary File 1
