# Supplementary material for: National Differences in Requirements for Ethical and Competent Authority Approval for a Multinational Vaccine Trial under the EU Directive 2001/20/EC
Source: Vaccines (Basel). 2015 Apr 14;3(2):263–92. doi: 10.3390/vaccines3020263 (PMC4494354; doi:10.3390/vaccines3020263)
Supplement: Supplementary File 1 [file vaccines-03-00263-s001.docx]

**Supplementary Materials**

**Table S1.** Overview of the required documentation to be submitted to the ethics committees in Finland, Hungary, The Netherlands, Norway and Slovenia in an application for a favourable opinion.

| **Documents** | **Member States** | | | | |
| --- | --- | --- | --- | --- | --- |
|  | **Finland ^1^ [33]** | **Hungary [17,42]** | **The Netherlands [60,61,65]** | **Norway [62]** | **Slovenia [68]** |
| **Documents to be submitted in almost every Member State [13]** | | | | | |
| Signed cover letter |  | **X** | **X** |  | **X** |
| Signed application form | **X** | **X** | **X** | **X** |  |
| Clinical trial protocol | **X** | **X** | **X** | **X** | **X** |
| Summary of the protocol in the national language | **X** | **X** | **X** | **X** | **X** |
| Investigator’s brochure | **X** | **X** | **X** | **X** |  |
| Arrangements for recruitment of subjects | **X** | **X** | **X** | **X** | **X** |
| Subject information leaflet | **X** | **X** | **X** | **X** | **X** |
| Informed consent form | **X** | **X** | **X** | **X** | **X** |
| Description of the measures taken to safeguard the subject’s privacy and protection | **X** |  |  |  | **X** |
| Curriculum vitae and/or other relevant documents of the principal investigator | **X** | **X** | **X** | **X** | **X** |
| A description of facilities of the trial | **X** | **X** | **X** |  |  |
| A description of the provisions for indemnity or compensation in case of injury or death of trial subjects | **X** |  | **X** | **X** | **X** |
| A description of any insurance or indemnity to cover the liability of the sponsor and the investigator |  | **X** | **X** |  | **X** |
| Information about financial arrangements between the sponsor and the subjects and/or investigators | **X** | **X** | **X** | **X** | **X** |
| **Specific requested information** | | | | | |
| General | | | | | |
| List of competent authorities with the EU to which the application has been submitted and details on the decision |  | **X** | **X** |  |  |
| Copy of the ethics committee opinion in the Member States |  | **X** |  |  | **X** |
| Copy of the ethics committee opinion of other Member States |  |  | **X** |  |  |
| Copies of the assessment by other authorities like an advice or registration authority like the FDA or EMA |  |  | **X** |  |  |
| Information about the sponsor and, if applicable, the CRO |  |  |  |  | **X** |
| Letter of authorisation if the applicant is not the sponsor |  | **X** | **X** |  |  |
| Confirmation of the EudraCT number |  | **X** | **X** |  |  |
| If available peer review of the scientific value of the trial |  | **X** | **X** |  | **X** |

**Table S1.** *Cont.*

| **Documents** | **Member States** | | | | |
| --- | --- | --- | --- | --- | --- |
|  | **Finland ^1^ [33]** | **Hungary [17,42]** | **The Netherlands [60,61,65]** | **Norway [62]** | **Slovenia [68]** |
| **Specific requested information** | | | | | |
| General | | | | | |
| Statement by the principal investigator regarding conformity of the trial with research ethics | **X** | **X** |  |  | **X** |
| Statement by the head of the health institution or department regarding conformity of the trial with research ethics |  |  |  |  | **X** |
| Statement of admission of the trial by the head of the health institution |  | **X** |  |  | **X** |
| The composition and charter of the Data Safety and Monitoring Board |  |  | **X** |  |  |
| Protocol | | | | | |
| The exact age of the human subjects |  | **X** |  |  |  |
| Permission of the director of the hospital |  | **X** |  |  |  |
| Description of research biobanks |  |  |  | **X** |  |
| Aim and scientific rational, supported by a review of recent literature |  |  |  |  | **X** |
| Proposers’ own perception of ethical issues involved in the trial |  |  |  |  | **X** |
| IMP | | | | | |
| IMPD |  | **X** | **X** |  |  |
| Examples of the labels in the national language |  | **X** | **X** |  |  |
| Declaration of GMP status of active biological substances |  | **X** |  |  |  |
| Copy of the manufacturing authorisation if the IMP is manufactured in the EU |  | **X** | **X** |  |  |
| Declaration of the qualified person that the manufacturing site works in compliance with EU GMP |  | **X** |  |  |  |
| Copy of the importer authorisation |  | **X** | **X** |  |  |
| Analytical certificate of the IMP |  | **X** |  |  |  |
| If available applicable authorizations to cover trials or products with special characteristics |  | **X** |  |  |  |
| If applicable a Transmitting Animal Spongiform Encephalopathy certificate |  | **X** |  |  |  |
| Certificates of qualified persons |  |  | **X** |  |  |
| A trading license if the IMPs are stored |  |  | **X** |  |  |
| If applicable viral safety studies |  | **X** |  |  |  |
| Sample in a quantity for a full analysis |  | **X** |  |  |  |
| If a placebo is used, a case specific justification of the necessity of the control group |  | **X** |  |  |  |

**Table S1.** *Cont.*

| **Documents** | **Member States** | | | | |
| --- | --- | --- | --- | --- | --- |
|  | **Finland ^1^ [33]** | **Hungary [17,42]** | **The Netherlands [60,61,65]** | **Norway [62]** | **Slovenia [68]** |
| **Specific requested information** | | | | | |
| IMP | | | | | |
| If applicable the product information from the hospital pharmacist such as prescription request forms |  |  | **X** |  |  |
| Trial subjects | | | | | |
| Other information material that will be made available to potential subjects such as diaries | **X** |  | **X** | **X** |  |
| All questionnaires the human subjects should fill in during the research |  |  | **X** | **X** |  |
| If applicable, the patient card listing the research the human subject participates in with the contact  information for the sponsor and/or investigator |  |  | **X** |  |  |
| Statement by the patient that appropriate measures will be taken to prevent pregnancy in case of  research on patietns in fertile period with a risk of mutagenicity |  |  |  |  | **X** |
| Details about how the subject can contact the responsible doctor in case of emergency |  |  |  |  | **X** |
| A description of how the interest and right to proper medical care will be assured if a  control group will be involved |  |  |  |  | **X** |
| Trial site and investigators | | | | | |
| List of trials sites and investigators in the Member State | **X** |  | **X** |  |  |
| Information on the supporting staff |  | **X** |  |  |  |
| Curriculum vitae independent expert |  |  | **X** |  | **X** |
| Curriculum vitae coordinating investigator |  |  | **X** |  |  |
| Statement on the feasibility of the research in the national centre from the head of the department |  |  | **X** |  |  |
| Signed clinical trial agreement between the sponsor or financier with the investigator and/or institution |  |  | **X** |  |  |

^1^ The required documents to be submitted to an Ethics Committee in Finland are based on the operating procedure of TUKIJA. If a regional ethics committee is the ethics committee that has to give an opinion about a vaccine trial, information about the documents that has to be submitted to the regional ethics committee can be found on the website of the specific regional ethics committee. In general, the documents are essentially the same as the documents that has to be submitted to TUKIJA. However, an additional document that has to be submitted is the decision of TUKIJA on delegating the handling of the trial to the specific regional ethics committee.

**Table S2.** Overview of the required documentation to be submitted to the competent authorities in Finland, Hungary, The Netherlands, Norway and Slovenia in an application for competent authority approval.

| **Documents** | **Member States** | | | | |
| --- | --- | --- | --- | --- | --- |
|  | **Finland [75]** | **Hungary [17,42]** | **The Netherlands [60,61,65]** | **Norway [26]** | **Slovenia [90,91]** |
| **Documents to be submitted in almost every Member State [59]** | | | | | |
| Signed cover letter | **X** | **X** | **X** | **X** | **X** |
| Signed application form | **X** | **X** | **X** | **X** | **X** |
| Clinical trial protocol | **X** | **X** | **X** | **X** | **X** |
| Investigator’s brochure | **X** | **X** | **X** | **X** | **X** |
| IMPD | **X** | **X** | **X** | **X** | **X** |
| Copy of the ethics committee opinion in the Member States |  | **X** |  | **X** | **X** |
| Examples of the labels in the national language |  | **X** | **X** | **X** | **X** |
| Subject information leaflet | **X** | **X** | **X** |  | **X** |
| Informed consent form | **X** | **X** | **X** |  | **X** |
| Summary of the protocol in the national language |  | **X** | **X** |  | **X** |
| **Specific requested information** | | | | | |
| General | | | | | |
| Opinion of the Paediatric Committee if the clinical trial is part of a n agree paediatric investigation plan |  |  |  | **X** |  |
| Copy of the ethics committee opinion of other Member States |  |  | **X** |  |  |
| Copies of the assessment by other authorities like an advice or registration authority like the FDA or EMA |  |  | **X** | **X** |  |
| List of competent authorities with the EU to which the application has been submitted and details on the decision |  | **X** | **X** |  | **X** |
| Letter of authorisation if the applicant is not the sponsor | **X** | **X** | **X** |  |  |
| Confirmation of the EudraCT number |  | **X** | **X** |  |  |
| If available peer review of the scientific value of the trial |  | **X** | **X** |  |  |
| Statement by the principal investigator regarding conformity of the trial with research ethics |  | **X** |  |  | **X^1^** |
| Statement of admission of the trial by the head of the health institution |  | **X** |  |  |  |
| The composition and charter of the Data Safety and Monitoring Board |  |  | **X** |  |  |
| The preparedness in the event of complications (if not described in the protocol) |  |  |  | **X** |  |
| Completed KLPR-A form with data on the clinical trial |  |  |  |  | **X** |

**Table S2.** *Cont.*

| **Documents** | **Member States** | | | | |
| --- | --- | --- | --- | --- | --- |
|  | **Finland [75]** | **Hungary [17,42]** | **The Netherlands [60,61,65]** | **Norway [26]** | **Slovenia [90,91]** |
| **Specific requested information** | | | | | |
| Protocol | | | | | |
| The exact age of the human subjects |  | **X** |  |  |  |
| Permission of the director of the hospital |  | **X** |  |  |  |
| IMP | | | | | |
| Declaration of GMP status of active biological substances |  | **X** |  |  |  |
| Copy of the manufacturing authorisation if the IMP is manufactured in the EU |  | **X** | **X** |  | **X** |
| Declaration of the qualified person or a responsible person that the manufacturing site works in compliance with EU GMP |  | **X** |  |  | **X** |
| Copy of the importer authorisation |  | **X** | **X** |  | **X** |
| Analytical certificate of the IMP |  | **X** |  |  |  |
| If available applicable authorizations to cover trials or products with special characteristics |  | **X** |  |  |  |
| If applicable a Transmitting Animal Spongiform Encephalopathy certificate |  | **X** |  |  |  |
| Certificates of qualified persons |  |  | **X** |  |  |
| A trading license if the IMPs are stored |  |  | **X** |  |  |
| If applicable viral safety studies |  | **X** |  |  |  |
| Sample in a quantity for a full analysis |  | **X** |  |  |  |
| If a placebo is used, a case specific justification of the necessity of the control group |  | **X** |  |  |  |
| If applicable the product information from the hospital pharmacist such as prescription request forms |  |  | **X** |  |  |
| Trial subjects | | | | | |
| Other information material that will be made available to potential subjects such as diaries |  |  | **X** |  |  |
| All questionnaires the human subjects should fill in during the research |  |  | **X** |  |  |
| If applicable, the patient card listing the research the human subject participates in with the contact information for the sponsor and/or investigator |  |  | **X** |  |  |
| Arrangements for recruitment of subjects |  | **X** | **X** |  |  |

**Table S2.** *Cont.*

| **Documents** | **Member States** | | | | |
| --- | --- | --- | --- | --- | --- |
|  | **Finland [75]** | **Hungary [17,42]** | **The Netherlands [60,61,65]** | **Norway [26]** | **Slovenia [90,91]** |
| **Specific requested information** | | | | | |
| Trial site and investigators | | | | | |
| List of trials sites and investigators in the Member State | **X** |  | **X** |  |  |
| Information on the supporting staff |  | **X** |  |  |  |
| Curriculum vitae and/or other relevant documents of the principal investigator |  | **X** | **X** |  | **X** |
| Curriculum vitae independent expert |  |  | **X** |  |  |
| Curriculum vitae coordinating investigator |  |  | **X** |  |  |
| Curriculum vitae of the person responsible for the trial in Member State | **X** |  |  |  |  |
| Statement on the feasibility of the research in the national centre from the head of the department |  |  | **X** |  |  |
| A description of facilities of the trial |  | **X** | **X** |  |  |
| Signed clinical trial agreement between the sponsor or financier with the investigator and/or institution |  |  | **X** |  |  |
| A brief description of any unusual division of duties and responsibilities between the sponsor  and the person responsible for the trial | **X** |  |  |  |  |
| Completed KLPR-C form with the consent of the responsible person of the entity conducting the  trial to the appointment of the principal investigator and the use of premises, personnel and  equipment for conducting the clinical trial |  |  |  |  | **X** |
| Financial | | | | | |
| A description of the provisions for indemnity or compensation in case of injury or death of trial subjects |  |  | **X** |  |  |
| A description of any insurance or indemnity to cover the liability of the sponsor and the investigator |  | **X** | **X** | **X** | **X** |
| Information about financial arrangements between the sponsor and the subjects and/or investigators |  | **X** | **X** |  |  |
| Administrative fee |  |  |  |  | **X** |
| In case of fees, proof of payment | **X** |  |  |  | **X** |

^1^ Completed KLPRA-B form with the principal investigator’s statement which is available at http://www.jazmp.si/en/human_medicines/forms/.
